# Assessment of Environmental Demands of Age-Friendly Communities from Perspectives of Different Residential Groups: A Case of Wuhan, China

**DOI:** 10.3390/ijerph19159120

**Published:** 2022-07-26

**Authors:** Jintao Li, Yan Dai, Cynthia Changxin Wang, Jun Sun

**Affiliations:** 1 School of Civil Engineering, Architecture and Environment, Hubei University of Technology, Wuhan 430068, China; jintaohbut@hbut.edu.cn (J.L.); daiyan_xyz@163.com (Y.D.); 2 Innovation Demonstration Base of Ecological Environment Geotechnical and Ecological Restoration of Rivers and Lakes, Hubei University of Technology, Wuhan 430068, China; 3 School of Built Environment, University of New South Wales, Sydney 2052, Australia; cynthia.wang@unsw.edu.au; 4 School of Civil and Hydraulic Engineering, Huazhong University of Science and Technology, Wuhan 430074, China

**Keywords:** age-friendly community environment, residential demand, differences, nonparametric test

## Abstract

Age-friendly communities (AFCs) are an important measure for fostering active aging. The key to achieving an age-friendly living environment is to construct or renovate it according to the residents’ demands. To date, very few studies have attempted to delve into the AFCs’ environmental demand from different groups’ perspectives. Based on the theory of place attachment, the aim of this paper is to explore the demand diversity of different groups for the AFC environment. This study employs the nonparametric test and the Ordinal Priority Approach (OPA) to investigate the demands from the residents’ perspectives, and is enhanced by incorporating experts’ opinions. The empirical analysis shows that residents have a high level of demand for the physical environment (indoor and outdoor) and social environment (community services and social participation). At the same time, experts advocate the importance of using digital technologies to support people aged 45–65 who have higher requirements for a community environment than older adults. The findings also show that other backgrounds, such as gender, living arrangements, and year of the community establishment, lead to different demands. However, the impact of residents’ education level, occupation, income, and self-care ability on the environmental demands is low. Based on the research findings, the paper provides some practical suggestions for the future design and development of AFCs.

## 1. Introduction

The World Health Organization (WHO) first proposed the concept of “Age-Friendly Communities (AFCs)” in 2005 and launched the AFCs project worldwide [1]. To date, the WHO Global Network for AFCs involves a total of 1333 cities and communities in 47 countries, of which only one is in mainland China [2]. China’s 2020 census data showed that the population aged 60 and above is 268 million, accounting for 18.87% of the country’s population [3]. Actively responding to population aging has been promoted as a national strategy by the Chinese government, and the creation of AFCs is an innovative carrier for the implementation of this strategy. China’s 14th five-year plan (2021–2025) stated that the demands of Chinese older people are changing from survival to development of quality of life. In particular, the “baby boomers” born after 1962 have gradually entered the aging stage. They have generally accumulated some wealth, can afford higher living expenses, and have more expectations and requirements of the community environment.

According to the existing literature, in China’s urban communities, more than 60% of older people live independently (alone or with a spouse). Even for residents aged 80 and above, the proportion of independent living is as high as 48%. With the decline of mobility and skills, they require a more supportive community environment. More than 60% of Chinese seniors spend their time in communities. Exercising outdoors, chatting with other residents, and taking grandchildren out to play are high-frequency activities for them [4]. Specifically, the daily activities that various age groups often engage in are different, and the corresponding needs of the community environment are also different. For example, the demand by younger residents for sports and fitness venues is higher than that for older residents. Older residents also have a strong demand for community medical services, with health monitoring and home-based rehabilitation after illness, being the most desired community medical services, followed by medicine delivery services and family doctor visits. Furthermore, the daily lives of Chinese people are increasingly dependent on technology. Due to poor adaptability to technology, older adults are facing difficulties in using digital products and platforms [5,6,7,8,9].

There is a large body of literature on the AFC environment from the perspective of older adults in developed countries, which has explored the factors influencing the happiness of the aging population from the viewpoint of the physical and social environment [10,11]. There are also some studies exploring the influencing factors of active aging in low-income countries [12]. In 2009, China launched a pilot project to build age-friendly cities and livable communities for aged people. In recent years, there have been multiple studies on the demand for community aged care services in China [13]. A series of papers have focused on the effect of the community environment on residents’ satisfaction and quality of life [14,15], and how to promote age-friendly e-participation in the digital era [16]. However, few studies have explored AFCs from the perspective of the demands of different groups. Existing research on the AFCs environment is insufficient in several respects. Firstly, the research on AFC in developed countries is relatively mature, while Chinese knowledge of AFCs is in its infancy. However, combining the analysis of the domestic situation and the experience of developed countries is helpful in promoting the construction and development of China’s AFCs. Secondly, existing studies on AFCs in China only described the needs of the aged people in general and did not examine the requirements and needs of different groups. Thirdly, no study has incorporated the perspectives of older people in the communities and matched them with the opinion of experts on aged care services.

This paper aims to fill these knowledge gaps and explore the varying demands on the AFCs’ environment from people with different backgrounds. A random sample survey of 438 tenants in Wuhan was conducted, and non-parametric tests and OPA are employed to explore the AFCs’ varying demands by different residential groups. The following areas will be explored in this investigation: (i) the community environmental indicators that reflect important residents’ needs; (ii) the range of opinions from the perspectives of the experts and residents; and (iii) the different demands that arose from the residents of different backgrounds. These findings not only provide relevance to multiple perspectives on AFCs, but also point to directions for future AFC development.

## 2. Literature Review and Conceptual Framework

### 2.1. The Age-Friendly Community Environment

Age-friendly cities and communities aim to provide a livable and lifelong-supporting environment that meets older adults’ families, communities, and society’s needs [17,18]. In light of the existing literature, the environment is defined as the physical and social environment that accommodates individuals [19]. The process of interaction between the aged and their physical–social environment depends on a variety of parameters, such as personal characteristics (gender, age, education, occupation, living arrangements, and self-care ability); the physical environment (housing, neighborhoods, parks, barriers, and care facilities); and the social environment (social inclusion and social support) [20,21]. The interaction of assisted living and health-related technology systems also has an increasing impact on the quality of life of older persons [22].

Early research on AFCs highlighted the physical environment [23]. A supportive physical environment becomes essential for older adults when they suffer from increased physical dysfunction and limited mobility [24]. Yu et al. [15] argued that improving the physical environment can help reduce pedestrian hazards on the road, enhance perimeter accessibility, and encourage outdoor activities. Some countries have issued design guidelines, such as the Lifetime Homes Design Guide in the UK and Australia’s Livable Housing Design Guidelines, to provide a comfortable living environment for the aged [25]. Sweden has also attempted to eliminate physical environmental barriers with housing renovations [26]. Lui et al. suggested that a supportive social environment is as important as the physical environment in improving well-being in late life [27]. A positive social environment can provide much support through education, employment and volunteering, and participation in community activities [28].

A large body of literature demonstrates that it is common for the elderly to feel lonely and depressed due to major changes in life, such as retirement, the death of loved ones, declining physical condition, and separation from society [29,30]. Participating in community activities helps to improve sleep, cognitive functions, and mental health in the elderly [31], and reduce their loneliness and isolation [32]. In Japan, many communities implemented community-based social innovation (CBSI), which facilitates resident interaction by organizing group fitness activities and socio-cultural activities [33]. A European project called Homes4Life is developing Smart AFCs, which are not just related to the physical environment, but also concern the personal and social domains [34]. The main principles behind promoting active aging through an age-friendly environment have spread around the world, though some questions about the AFCs’ environment have not been addressed. For example, does the concept of AFCs take all older persons into account? Do AFCs cater to the diverse needs of seniors [35]? This research attempts to explore these questions.

### 2.2. Age-Friendly Community Environmental Demands Indicators

Many researchers have confirmed that the interior environments of rooms have an impact on elders’ subjective well-being. Dilapidated houses or lack of internal amenities are harmful to the aged [36]. Chau and Jamei pointed out that older persons’ willingness to spend time outdoors would increase if the outdoor environment were conducive to the elderly [25] since a safe and accessible outdoor environment can enhance the independence and health of the elderly [37]. Examples are more seating and lighting in public spaces, unobstructed and wheelchair-accessible sidewalks, accessibility to transportation systems, and public space [38].

Menec et al. argued that a positive social environment helps people feel a sense of belonging, especially the aged [39]. Spain offered long-term care services for the elders according to an established protocol of care. The authorities deployed different health resources such as hospitals, palliative care, and home care, as well as social resources like day centers, supervised housing, and technical support [40]. Japan established Community Gathering Spaces (CGSs) as a social platform for older people and created a social network in the community to prevent older adults’ households from becoming socially isolated [41,42]. Numerous studies have shown that elders’ participation in social or community activities can reduce feelings of disconnection and increase self-confidence and respect. Volunteering or paid employment also has a statistically significant influence on active aging [43,44].

Intelligent aged care is gradually maturing due to the development of technology. Information sharing platforms can optimize the user experience and help seniors filter misinformation [5]. The demands of older people for intelligent aging products (e.g., smart bracelets, smartwatches, smart crutches, and smart call badges) are increasing [44]. However, older adults’ ability to adapt to modern digital life is weak, and many of them cannot access the Internet, download APPs, or obtain the required information digitally, which means older people cannot enjoy the convenience brought by digital and intelligent services [6,7,8,16]. According to the Chinese National Bureau of Statistics, 13.5% of China’s population was over 65 in 2020. By 2020, China’s Internet users over 60 years old comprised only 6.7% of the total number of Internet users. On the user side, due to the weakness of digital skills, the proportion of the elderly using search engines, APPs, and WeChat was significantly lower than that of young people in China. Among them, the proportion of the elderly using search engines was 4.4%, and less than 1/6 of the non-elderly Internet users. The number of mobile APPs per capita for the elderly was 37, which was only 44% of the number of young Internet users aged 20–29 (84 per capita). The proportion of the elderly using WeChat was 26.2%, and is less than 1/3 of the non-elderly users (88.9%) [9]. This dilemma of the “digital divide” has caused challenges in using intelligent devices to support aged care in society.

### 2.3. Different Demands of an Age-Friendly Community Environment

There are significantly different demands for community housing, social activity, and services among different age groups [11]. Younger seniors have stronger demands for benches, accessibility, and fitness equipment in the community [45]. Walsh et al. investigated the expectations of residents aged 55–64 on their needs for future community living environments and found that participants would consider the quality of life only after basic needs, such as housing, had been met [46]. Older persons in different types of communities may also have different perceptions of what is age-friendliness [21]. The greater the number of older people in the community, the more age-friendly the community needs to be, especially in terms of social participation, communication, and access to information [47]. However, people who are socially and economically disadvantaged have a poor self-assessment of age-friendliness [46]. A series of papers has shown that elder adults’ awareness of environmental friendliness varies according to their physical function, health, family, and income, in consistence with the findings of the general public [48,49,50]. For instance, Naah et al. indicated that older people who live in communities with higher environmental standards, healthy lifestyles, higher education, and have partners, for example, are more active than others [51].

At present, the construction of elderly care service facilities in China differs depending on whether the focus is meeting survival needs or the development needs of the aged. To date, community public facilities can meet the survival needs of older people. However, in terms of community care services and community participation, facilities cannot meet the general development needs of the aged population [5]. In addition, ongoing facility management and support are crucial to satisfy residents’ needs [52], but there is a lack of continuous support for the aged who wish to use digital aged care products [53]. In summary, the current community care facilities are not “user friendly” for older adults, and there is an obvious gap between the level of community facilities and the expectations of older people. Previous research has enriched our understanding of the AFCs, but few studies have explored the environment of AFCs and their ability to meet the varying needs of residents. In this study, the varied demands of different elderly participants connected with the community environment will be investigated to fill the above knowledge gap.

### 2.4. Conceptual Framework and Hypotheses Development

Place attachment (PA) is a one-way emotional bond between people and their surrounding environment [54], including a sense of belonging, self-expression, and psychological security [55]. It is a strong emotional orientation produced by peoples’ communication with society for many years in the process of long-term living, that is, peoples’ expectation of a place or a mutually beneficial support system [56].

The concept of PA was formally put forward by Williams and Roggenbuck in 1989, that is, a connection between people and places based on emotion, cognition, and practice [57]. Scannell et al. summarized the previous scattered concepts and proposed a three-dimensional framework to explain PA. It is the people attached to a certain place, the emotions generated in the process of people connecting with the place, and the places with different characteristics gaining people’s attachment [58]. The place dimension is also subdivided into the physical and social environment, which explains in more detail the places involved in people’s attachment and the differences between those places. Lewicka further explains PA as representing a touchable and real physical foundation, and it is also an entity with a social dimension. Thus, attachment can be the dependence on a local tangible asset, or it can be the adherence to interrelated social factors [59].

The concepts of PA and “community attachment” often overlap. The physical environment of a place is usually regarded as a container of social processes rather than an independent research object [60]. Community plays a significant role in the formation of PA, which leads to the connection and close interaction between residents and their environment. Anderson and Fulton found that the higher the residents’ level of PA, the higher their political and social participation in the community [61]. PA to the community is explained by various structures. These structures include the sense of place belonging, where people feel they are members of an environment, which builds a strong connection with home; a locational familiarity that forms the environmental memory attached to the place and its neighborhood, which maintains a person’s emotional connection with the surrounding environment [62]. Social participation can also promote PA and personal mobility. It can also strengthen feelings of familiarity and positive emotional ties between older persons and their neighbors [63].

**Figure 1:**
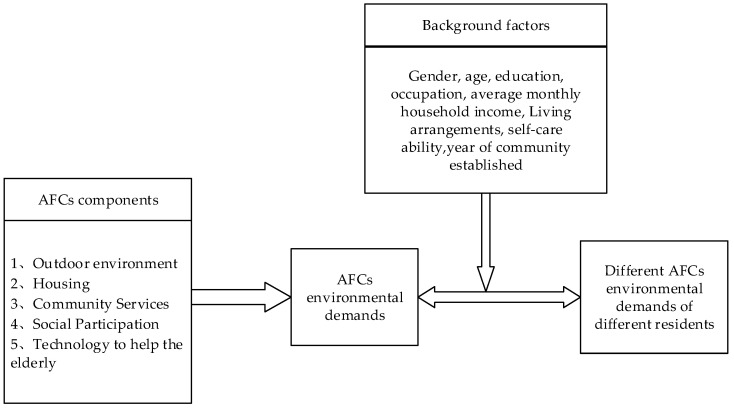
A conceptual framework for AFCs’ environmental demands.

According to the PA and the abovementioned literature, AFCs environmental components are identified, and a conceptual framework for the AFCs environment demands is constructed, as shown in Figure 1.

In Figure 1, the physical environment (outdoor environment and housing) and social environment (community services and social participation) are used to explore residents’ demands for the AFCs environment. Meanwhile, with the wide application of technology in elderly care in recent years, we add technology-supported care into the survey to understand the residents’ views of and demand for technology. Therefore, five main aspects of AFCs environmental components are established. Meanwhile, the environmental demands of residents may be affected by their socio-demographic nature (gender, age, education, occupation, income, living arrangements, self-care ability, and age of community) [49,50,51]. To study the differences in AFC’s environmental needs of residents with different backgrounds, the following hypotheses are established:

**H1**.  *There are significant differences between the demands of men and women in the AFCs environment.*

**H2**.  *The environmental demands of AFCs vary significantly with the age of residents.*

**H3**.  *Education has a significant impact on the environmental demands of residents in AFCs.*

**H4**.  *Different occupations lead to significantly different demands of residents in the AFCs environment.*

**H5**.  *Different incomes lead to a significant difference in residents’ demands in the AFCs environment.*

**H6**.  *Living arrangements have a significant impact on the environmental demands of residents in AFCs (in this paper, living arrangements refer to whether the person lives alone, with a partner, or with other family members).*

**H7**.  *There are significant differences in the environmental demands of AFCs in terms of self-care ability.*

**H8**.  *The establishment year of the community also leads to significant differences in residents’ environmental demands for AFCs.*

## 3. Data Collection and Analysis Method

### 3.1. Questionnaire Design

This study is based on the theory of PA and analysis of a considerable quantity of the existing literature, WHO guidelines and China’s policy documents on the AFCs environment. In total, five first-level indicators and 21s-level indicators of environmental demand are identified, as shown in Table 1. To gain insights into the degree of individual demands, we asked respondents to rate the 21s-level indicators on a five-point Likert scale ranging from 1 (“very slightly” or “not at all” important) to 5 (“extremely” important).

In this study, a questionnaire survey was conducted among mid to old-aged people over 45 to investigate the differences in environmental needs due to different ages. Existing literature indicates that the definition of middle and old age is not clear-cut. However, most studies agree that the mid-40s is a starting point for middle age, and 65 has long been used to define the beginning of “old age” [64,65,66]. Therefore, in the design of this questionnaire, the age of the respondents starts from 45 years old, and the groups are subdivided by every 10 years above that age. From this, we analyze the differences in community environmental needs resulting from age differences.

**Table 1:** Environmental demand indicators experienced by AFCs.

First-Level Indicators	Second-Level Indicators	Sources
Outdoor environment	Community safety	Kim et al., 2021 [67]
	Community public facilities	Hu et al., 2021 [5]
	Community sanitation	Yu et al., 2021 [15]
	Public transportation station accessibility	Xie et al., 2018 [14]
	Accessibility to frequently visited places	Xie et al., 2018 [14]
housing	House decoration	Kim et al., 2021 [67]
	Convenient facilities inside the room	Kim et al., 2021 [67]
	Inspection and repair of household equipment	Yu et al., 2021 [15]
	Barrier-free construction and renovation	Del et al., 2021 [68]
Community services	Home care	Iglesias et al., 2021 [40]
	Medical and health care institutions	Xiang et al., 2020 [69]
	Health courses	Steels et al., 2015 [70]
	Information acquisition and regular visits	Yu et al., 2021 [15]
	Education and legal aid	Del et al., 2021 [68]
Social participation	Social activities	Yu et al., 2021 [15]
	Occupation opportunities	Steels et al., 2015 [70]
	Decision-making participation	Plouffe & Kalache, 2010 [71]
	Senior university study sites	Yu et al., 2021 [15]
Technology to help older adults	Aging information platform	van Hoof & Marston, 2021 [72]
	Smart product learning	Chen et al., 2021 [16]
	Intelligent aging products	Chen et al., 2021 [16]

### 3.2. Data Collection

The questionnaire survey was carried out in Wuhan, China, from November to December 2021. Before the survey, the communities in Wuhan were divided into three categories according to the year of construction, those built before 2000, during 2001–2010, and after 2010. Five communities of each type were selected for a random survey. This survey adopts the random sampling method, with a combination of offline and online surveys. We conducted the offline survey on the residents in the communities that allow visitors to enter, and at every fifth house. Due to the control measure during COVID-19, some communities were inaccessible, so we conducted an online survey on the residents of these communities. We shared the link of the questionnaire to the communities’ WeChat group, and invited residents in every fifth house to participate in the survey. Each household only filled in one questionnaire. In each sampling area, only residents who were 45 years old and above were invited. A total of 438 questionnaires were collected, of which 409 were valid.

We also invited three experts in the fields of aged health care, gene anti-aging, and general health, to rank the indicators to understand the demand tendency from their specialist perspectives.

### 3.3. Analysis Method

#### 3.3.1. Non-Parametric Statistics

Non-parametric models do not make assumptions about the model structure and do not rely on the distribution of variables [63]. In the modeling process, it can estimate the changing variables and the complicated relationships between variables. There are various methods of non-parametric tests. This study uses two independent samples of Mann–Whitney U to test whether there are significant differences in the needs of respondents of different genders. The Kruskal–Wallis method is used to test whether there are significant differences in the demands for AFCs environments with different ages, education, occupation, and other backgrounds.

#### 3.3.2. OPA or Ordinal Priority Approach

OPA is a multi-criteria decision-making method (MCDM) that has been developed in recent years. A significant advantage of this method is that it does not require pairwise comparisons, data normalization, and completeness. Another advantage is that experts can only comment and rank the index attributes for which they have sufficient knowledge and experience. The method supports incomplete data and group decision-making as well as the same-level ranking of different indicators [73,74]. OPA takes sequential data as inputs and in group decision-making, experts’ opinion is ranked according to their level of experience. It is effective to have more than one expert in the group decision-making process.

In this study, according to the working time and professionalism of the three invited experts, we rank Expert 1 first. Experts 2 and 3 are ranked joint second owing to their working hours and experience in this field being similar. The linear model is established according to the collected data, and can be solved by using LINGO, MATLAB, or OPA solver, to obtain the weighting of each expert and index, and then determine the demand priority.

## 4. Results

### 4.1. Reliability and Validity Tests

In this study, the results of the reliability test show that Cronbach’s α coefficient is 0.908, demonstrating good statistical reliability. The results of the sample suitability test show a KMO value of 0.896 with a significance of 0.000, which means that the exploratory factor analysis can continue to examine validity. The cumulative variance contribution is 65.213%, which indicates that the data is relatively reliable.

### 4.2. Descriptive Statistics

#### 4.2.1. Description of Socio-Demographic Characteristics

The socio-demographic characteristics of the respondents are listed in Table 2, indicating that the participants have a wide range of backgrounds.

#### 4.2.2. Expert Ranking

The three experts’ rankings for the first-level and second-level indicators are shown in Table 3 and Table 4, respectively.

### 4.3. Age-Friendly Community Environment Demands

#### 4.3.1. Residents’ Demand for the Age-Friendly Community Environment

The mean value and ranking of the indicators of the overall demands are shown in Table 5. The higher the mean value, the higher the demand. The smaller the ranking value, the more important the indicator. As shown in Table 5, the mean values of the outdoor environment and housing in the first-level indicator are almost the same. Social participation and community service are slightly lower than the former two, and the elders’ demand for technology is the lowest. Residents’ demand for some second-level indicators, such as community public facilities, maintenance of household equipment, education and legal aid, and social activities, is higher than other secondary indicators.

#### 4.3.2. Expert Ranking of Environmental Indicators for AFCs

OPA solver is used to analyzing the ranking of experts on requirements and weights for each index and the results of the analysis are shown in Table 6. The smaller the ranking, the more important the indicators are. Housing and outdoor environment are the most important, followed by technology to help older persons, and social engagement and community service are the lowest. Concerning the second-level indicators, experts believe that convenient facilities inside the room, social activities, and aging information platforms are more important than other indicators.

### 4.4. Differential Impact of Residents’ Background Characteristics on Demands

A normality test is carried out for the collected data and the results are shown in Table 7. The significance of all data is less than 0.05, so the data is not normally distributed.

#### 4.4.1. Consistency Test among Respondents

Kendall’s consistency test is used to measure the degree of consistency of the residents’ indicator scores. As shown in Table 8, Kendall’s consistency coefficient (W) for the primary indicator scores is 0.041, and the secondary indicator coefficients (W) are 0.014, 0.026, 0.119, 0.074, and 0.156. The significance of all items is 0, but the calculated Kendall coefficient values are small, which means the level of consistency is low. Then, the Mann–Whitney U test is used to verify the impact of gender on demand, and the Kruskal–Wallis test is used to verify the impact of age, education level, occupation, and other backgrounds on demand.

#### 4.4.2. Mann–Whitney U Test

The results of the Mann–Whitney U test are shown in Table 9. The larger the mean value in the table, the stronger the demand of residents. The *p*-value of the secondary indicators listed is less than 0.05, indicating that at the 5% confidence level, residents’ demands for these indicators vary with gender. This result supports the first hypothesis (H1). IBM SPSS statistics can directly export Z values, but do not provide size effect X statistics, that is, the r-value. Equation (1) can be used to calculate the *r*-value (*r* = 0.1 for small effects, *r* = 0.3 for medium effects, *r* = 0.5 for large effects; r takes the absolute value). The value of *r* in Table 9 indicates that the influence of the demand indicators has a medium to large effect.

(1) $$r=Z/\sqrt{N},$$

where N is the total number of respondents.

#### 4.4.3. Kruskal–Wallis Test

The Kruskal–Wallis test results of the first-level indicators are shown in Table 10. A *p*-value less than 0.05 indicates that different groups have differences in demands, but it can only indicate that for each indicator. There are significant variations between at least two groups and their demands. To understand the different residents’ demands against the indicators in more detail, the second-level indicators are analyzed, and the results are given in Table 11. It shows that there are significant demand differences among residents of different ages. This finding strongly confirms the second hypothesis (H2): younger people have greater expressed demands for most indicators than older adults, and residents’ demands tend to decline with age.

The Kruskal–Wallis test results for demand differences for other residents’ backgrounds are shown in Table 12. It can be seen that different living styles and the year of community establishment lead to differences in residents’ needs for some indicators, which partially confirms hypotheses H6 and H8. Aged living alone leads to greater needs for the community environment than those already with companions. The longer the community is established, the greater the residents’ demand for the physical environment, especially in terms of community public facilities, convenient facilities inside the room, and construction and renovation for better accessibility. In addition, different income and physical conditions also lead to differences in demand on several other indicators, but not significantly so. The results do not fully support H3 and H4. Education level and occupation can hardly lead to differences in demand, therefore hypotheses H5 and H7 are rejected.

## 5. Discussions

### 5.1. Age-Friendly Community Environment Demands

Table 5 shows that the residents’ demand for a desirable physical environment is the strongest with the highest mean value for first-level indicators of outdoor environment and housing, followed by the demand for social participation and community services. This finding is consistent with the conclusions made by Lai et al. [75], which highlighted that in the process of constructing AFCs, physical barriers will hinder or prevent the social activities of older people and that the construction of the physical environment should take precedence over the social environment. The research indicates that maintaining the basic function of the physical environment is of the highest importance, and this is reflected by the high mean values for four second-level indicators including community public facilities, community sanitation, domestic equipment maintenance and repair, and medical and health care institutions.

Among second-level indicators, public transportation, accessibility of frequently visited places, and convenient facilities inside rooms are considered moderately important, which reveals that most of the residents in the survey have relatively good mobility and, therefore, these items are not in high demand. This finding is different from the findings of Yu et al. [76], who pointed out that transportation is the most important aspect of the physical environment. Obviously, this difference may be due to different urban development and traffic conditions.

House decoration receives a low mean value, and this demonstrates that older residents more focus on the essential physical components of their living environment, and see decorative items are less important and optional. Compared with house decoration, residents have higher requirements for indoor facilities and barrier-free construction and renovation. Simple housing renovation, such as installation of lighting and handrails in bathrooms, can greatly improve the daily activity ability and mental health of the aged [77].

While social participation is always indispensable for aged residents, in this research, older people’s demand for job opportunities is not high. A similar finding was reported by previous researchers [78], who indicated that older Chinese people have a low rate of participation in paid work and volunteer services. In contrast to the previous finding that community services and residents’ satisfaction are not necessarily related [14], this research reveals a particularly high demand for some community services such as education and legal aid, which shows that the aged have a good awareness of the importance of understanding and in compliance of the law, and liability prevention. This demand is consistent with the situation in China as the country has been improving its legal system and services to the public.

It is found that residents’ interest in in-home care is low, which may be related to the small number of aged care service providers in China and the varying service standard and procedures of these service providers. The demand for smart technologies to support the aged is also low. This also reflects that the Chinese market of digital products for the aged is immature, and the technologies’ degree of aging adaptation is low.

Compared with the above findings from the survey of residents, experts share the same opinion that the physical environment is the most important as the same first-level indicators (outdoor environment and housing) are ranked the highest, as shown in Table 6. However, the importance of technology is regarded as greater than the social environment according to the experts. They point out that extensive use of technologies is inevitable in future aged care services, and older people’s demand for technology-assisted aging products and services will increase. Experts have also emphasized the importance of medical and healthcare institutions. Recent studies have supported their opinion that a new model of “embedded smart aged-care facility” has emerged in China, which can provide services for specific groups of older people according to their varying demands [44].

### 5.2. Different Demands of Residents of an Age-Friendly Community Environment

#### 5.2.1. Difference in Demand by Gender

Table 9 compares the demand of men and women, and it shows that generally, women have a higher mean value than men for most of the indicators, which shows that women pay more attention to the community environment than men. This is consistent with Del et al. [68]’s study, which concluded that women have higher requirements for housing and social participation than men. More specifically, women have high demands for community sanitation, and domestic equipment maintenance and repair. This reflects the nature of the gender difference, as women have a higher hygienic requirement and need more assistance in repairing household items.

Women show stronger demand for social participation and education. They like to participate in social activities and learn new knowledge and skills in dedicated aged education programs. While men’s demand for social activities and education is not low, they have a much higher demand for medical and health care institutions and accessible facilities than women. This may reflect the fact that in general, men’s health status is not as good as women’s, and they need more medical care and support in old age, while women seek more intellectual-related social activities, e.g., attending educational courses for the aged.

Neither men nor women have much interest in learning about technology and smart products, and the mean scores for this indicator are significantly lower than other indicators.

#### 5.2.2. Differences in Demands by Age

The results in Table 10 and Table 11 show how the demands of residents vary with the different age groups. In general, the mean value for all indicators decreases with age. The demand for community environments for people aged 45–55 and 56–65 is higher than for people aged 66 and above. The purpose of this study to cover people aged 45–55 and 56–65 is to learn about their expectations for the AFCs environment, and the future direction of AFCs’ development can be established. The results of this study reveal that the current physical and social environments of the community are still far from the ideal as expressed by residents, especially for people aged 45–55 and 56–65. It is worth noting that people aged 45–55 and 56–65 have a higher demand for smart products than those aged 66 and above, which means that they have marked expectations for future technology-supported care, and this supports the experts’ opinion that technology-supported aged care will be in demand in the future.

According to Table 10, there are significant differences in the needs of different age groups for each first-level indicator. Residents aged 45–55 and 56–65 have a high demand for the outdoor environment and social participation, while the needs of people above 66 are focused more on housing and community services. This reflects that the demands of aged people will change when they are getting older, as their health status and mobility change.

The differences in demand for second-level indicators of age are further studied, as shown in Table 11. Residents aged 45–55 have a higher demand for all indicators except house decoration and home care than other age groups, suggesting that they have a higher demand for the future community environment. People aged 56–65 have reached retirement age, and they have a strong demand for social activities and health education programs. This finding aligns with the study by Cao et al. [43], who pointed out that the dependence of people upon the community environment will increase after they retire when they no longer have close ties to the work environment. They need to participate in social activities or health classes to spend time and find companionship. Residents who are 66 years old and above have significantly lower needs for most indicators than residents under 66, except for barrier-free construction and renovation and home care.

Residents of 45–55, 56–65, and 66–75 have a relatively high demand for social activities, although the demand decreases with age. This finding is consistent with a large number of previous studies [24,30,79] that point out that social participation can promote active aging and reduce loneliness: elders cannot live a normal life without social participation. For people aged 75 and above, their demands reduce significantly, especially for three indicators: barrier-free construction and renovation, home care, and medical and health care institutions. This is because the physical function and self-care ability of this age group have significantly decreased, and their demand reduces to basic needs and assistance to maintain a normal life.

#### 5.2.3. Difference in Demands of Other Backgrounds

Table 12 compares the demand of the aged people in different living arrangements and living in a community that was constructed in different eras. It shows that the aged who live alone have higher demands on their community environment than those who live with their partners or children. The finding supports the study by Chau and Jamei [25], who reported that the aged who live alone or lost a partner are more likely to suffer from loneliness, and therefore require more social support. It is also consistent with previous studies examined in this research that loneliness leads to great dependence on the community environment [80]. As mentioned earlier, the number of aged people living alone in China is increasing, and building an age-friendly environment is essential for them to maintain their normal life.

In research on community satisfaction, Yu et al. [76] found that most residents are more satisfied with the newly built community environment than the old ones. Similarly, this research found that residents in old communities have higher demands for the physical environment than in newer ones, as shown in Table 12. In particular, the residents of the old community have a strong willingness to retrofit community public facilities and higher demand for accessible facilities and renovation. From the perspective of many aged residents, they are more willing to “make do” in the old house than take the risk and effort of moving to a new community, where they will face the challenges of adapting to the new environment [58,81]. Consequently, it is important to improve existing communities’ facilities and services by renovation and retrofitting, to meet aged people’s needs.

## 6. Conclusions

### 6.1. A Summary of Research Findings

AFCs are an important initiative in China to cope with the aging population, and the community environment is vital for older adults’ living standards and quality of life. This study analyzes differences in community environmental demands from the perspective of several different groups, and the following conclusions were obtained:

First, residents’ highest demand is for the physical environment, followed by the social environment, and technological demand by elders is the lowest. However, experts emphasize the importance of adopting technologies to help the aged. Residents’ demands for public facilities and social activities are generally high and virtually all participants did not show great interest in in-home care and medical institutions.

Second, the demands of people aged 45–65 are greater than those of older persons for most indicators, and the demands tend to decline with age. In this investigation, people aged 45–65 have a high demand for public facilities and social activities, while accessible buildings and social activities are more important for older persons.

Finally, this study also reveals that some backgrounds such as gender, living arrangements, and year of community establishment also contribute to demand differences. Residents who are women, living alone, and residing in long-established communities have relatively high demand on the community environment. In the process of future community construction, the special demands of these groups should be taken into account to ensure their safety and convenience in community life.

### 6.2. Practical Implications

Based on the above conclusions some practical implications can be derived:

First of all, the construction of the AFCs’ environment should meet the primary needs of residents. Therefore, improving the physical and social environments is the key point, especially in providing community public facilities and social activities. Future AFCs need to satisfy older people’s demand for accessible facilities and provide more opportunities for residents to socialize, e.g., by creating an age-friendly social platform and organizing regular social activities. On the other hand, forward-planning is needed to consider the needs of the current younger residents.

Secondly, technology-supported care is an inevitable trend in the future, although most elderly people in this study did not show great interest in this area. The government could advocate the integration of digital facilities into the daily life of the aged, for example, by providing incentives to encourage the development of digital aged care products and train professional service personnel. If necessary, policies could be established for increasing subsidies for intelligent aged care products and providing tax relief for relevant enterprises. Meanwhile, the community can carry out training programs for the implementation of intelligent products to improve older adults’ ability to use the products.

Finally, the government can gradually change the public’s stereotypical image of an aging lifestyle and promote a variety of modern age-friendly community services. These might include community recreational activities, digital and internet-enabled aged care services, and an integrated cultural and health care model for older adults.

### 6.3. Research Limitations and Future Directions

In this study, residents’ demands of the AFCs’ community environment are explored from a multifaceted perspective. The findings provide an in-depth understanding of elders’ needs for the construction of future AFCs. However, there are still several limitations. First, this survey was conducted in Wuhan, China. Residents in other cities may have different needs for the AFCs’ environment due to different urban development conditions and the local natural environment. Future research can expand the scope of the investigation and prepare a more comprehensive investigation of people’s environmental needs in different cities. Second, the investigation of older adults who are in extreme situations such as lying-in bed at home or in nursing homes, may not be adequate due to the COVID-19 controls over mobility in the community. Further research can investigate groups that were not covered in this investigation. Despite these limitations, this study provides an extensive overview of the demands on the AFCs’ environment by different resident groups and can help the government make informed decisions on the future design and construction of age-friendly communities.

## Figures and Tables

**Table 2:** Description of socio-demographic characteristics.

Characteristics	Attributes	Number	Percentage (%)
Gender	Men	204	49.9
	Women	205	50.1
Age	45–55 years old	143	35.0
	56–65 years old	121	29.6
	66–75 years old	105	25.7
	76 years old and above	40	9.8
Education	Elementary school and below	71	17.4
	Junior high school	146	35.7
	High school/junior high school	109	26.7
	College/undergraduate	83	20.3
Occupation	Government or career employees	59	14.4
	State-owned enterprise workers	45	11
	Private company employees	55	13.4
	Individual operators	84	20.5
	Unemployed	104	25.4
	Other	62	15.2
Average monthly family income	Less than 1000 RMB	40	9.8
	Less than 1000 RMB	60	14.7
	2001–3000 RMB	63	15.4
	3001–4000 RMB	83	20.3
	4001 RMB or more	163	39.9
Living arrangements	Living alone	72	17.6
	Living with spouse	154	37.7
	Living with children	65	15.9
	Living with spouse and children	118	28.9
Self-care ability	Fully self-care	264	64.5
	Mostly self-care	91	22.2
	Basic self-care	32	7.8
	Mostly unable to self-care	13	3.2
	Fully unable to self-care	9	2.2
Year of community establishment	Before 2000	139	34
	2001–2010	181	44.3
	After 2010	89	21.8

**Table 3:** The ranking results for the first-level indicators by experts.

Experts’ Ranking			
First-Level Indicators	Expert 1	Expert 2	Expert 3
Outdoor environment	4	1	1
Housing	1	2	3
Community services	2	4	4
Social participation	5	4	5
Technology to help older adults	3	3	2

**Table 4:** The ranking results for the second-level indicators by experts.

First-Level Indicators	Second-Level Indicators	Experts’ Ranking		
		Expert 1	Expert 2	Expert 3
Outdoor environment	Community safety	1	3	4
	Community public facilities	4	1	2
	Community sanitation	2	2	1
	Public transportation station accessibility	3	4	3
	Accessibility of frequently visited places	3	5	5
Housing	House decoration	4	4	4
	Convenient facilities inside the room	1	2	1
	Domestic equipment maintenance and repair	3	1	2
	Barrier-free construction and renovation	2	3	3
Community services	Home care	1	3	3
	Medical and health care institutions	2	2	1
	Health courses	3	1	4
	Information acquisition and regular visits	4	5	5
	Education and legal aid	5	4	2
Social participation	Social activities	1	1	1
	Occupation opportunities	2	2	3
	Decision-making participation	4	4	4
	Senior university study sites	3	3	2
Technology to help older adults	Aging information platform	1	2	2
	Smart product learning	2	1	1
	Intelligent aging products	3	3	3

**Table 5:** Mean and rank of environmental demands in AFCs.

First-Level Indicators	Mean	Ranking	Second-Level Indicators	Mean	Ranking
Outdoor environment	3.34	1			
			Community safety	3.27	3
			Community public facilities	3.53	1
			Community sanitation	3.43	2
			Public transportation station accessibility	3.26	4
			Accessibility of frequently visited places	3.24	5
Housing	3.33	2			
			House decoration	3.17	4
			Convenient facilities inside the room	3.39	2
			Domestic equipment maintenance and repair	3.5	1
			Barrier-free construction and renovation	3.29	3
Community services	3.27	4			
			Home care	2.72	5
			Medical and health care institutions	3.48	2
			Health courses	3.07	4
			Information acquisition and regular visits	3.47	3
			Education and legal aid	3.62	1
Social participation	3.3	3			
			Social activities	3.52	1
			Job opportunities	3.47	2
			Decision-making participation	2.99	4
			Senior university study sites	3.21	3
Technology to help older adults	2.98	5			
			Ageing information platform	3.21	2
			Smart product learning	3.33	1
			Intelligent aging products	2.4	3

**Table 6:** Weighting and ranks for the indicators tested with OPA.

First-Level Indicators (Weightage)	Ranking	Second-Level Indicators	Weightage	Ranking
Outdoor environment (0.274)	2	Community safety	0.274	2
		Community public facilities	0.334	1
		Community sanitation	0.170	4
		Public transportation station accessibility	0.044	5
		Accessibility to frequently visited places	0.178	3
Housing (0.334)	1	House decoration	0.062	4
		Convenient facilities inside the room	0.458	1
		Domestic equipment maintenance and repair	0.271	2
		Barrier-free construction and renovation	0.208	3
Community Services (0.170)	4	Home care	0.307	1
		Medical and health care institutions	0.307	1
		Health courses	0.215	2
		Information Acquisition and Regular Visits	0.065	4
		Education and legal aid	0.107	3
Social prticipation (0.044)	5	Social activities	0.521	1
		Job opportunities	0.240	2
		Decision-making participation	0.063	4
		Senior university study sites	0.177	3
Technology to help older adults (0.178)	3	Aging information platform	0.480	1
		Smart product learning	0.400	2
		Intelligent aging products	0.120	3

**Table 7:** Kolmogorov–Smirnov test results.

	Outdoor Environment	Housing	Community Services	Social Participation	Technology to Help the Older Adults
Test statistics	0.132	0.093	0.154	0.125	0.114
Asymptotic saliency (two-tailed)	0.000	0.000	0.000	0.000	0.000

**Table 8:** Kendall consistency test results.

		Case Number	Kendall’s Consistency Coefficient (W)	Freedoms	Asymptotic Significance
First-level indicators		409	0.041	4	0.000
Second-level indicators	Outdoor environment	409	0.014	4	0.000
	Housing	409	0.026	3	0.000
	Community services	409	0.119	4	0.000
	Social participation	409	0.074	3	0.000
	Technology to help older adults	409	0.156	2	0.000

**Table 9:** Mann–Whitney U test for AFCs’ environmental indicators in terms of gender.

	Mean		Z	*p*-Value	R
	Men	Women			
Community sanitation	3.25	3.60	−2.723	0.006	0.605
Domestic equipment maintenance and repair	3.31	3.68	−2.425	0.015	0.539
Barrier-free construction and renovation	3.48	3.10	−3.661	0.000	0.814
Medical and health care institutions	3.40	2.73	−5.14	0.000	1.142
Education and legal aid	3.49	3.76	−2.454	0.014	0.545
Social activities	3.32	3.71	−2.793	0.005	0.621
Senior university study sites	3.24	3.71	−3.308	0.001	0.735
Ageing information platform	3.05	3.37	−2.414	0.016	0.536
Smart product learning	2.28	2.51	−2.219	0.027	0.493

**Table 10:** Kruskal–Wallis test results for primary indicators.

		First-Level Indicators				
		Outdoor Environment	Housing	Community Services	Social Participation	Technology to Help Older Adults
Age	45–55 years old	3.85	3.63	3.58	3.82	3.34
	56–65 years old	3.61	3.41	3.51	3.30	3.30
	66–75 years old	3.60	3.05	3.46	2.83	3.00
	76 years old and above	2.62	3.04	3.27	2.67	2.52
	*p*-value	0.001	0.004	0.000	0.000	0.034
Living arrangements	Living alone	3.44	3.58	3.68	3.63	3.27
	Living with spouse	3.40	3.25	3.42	3.26	3.20
	Living with children	3.06	3.09	3.19	2.75	3.05
	Living with spouse and children	3.11	3.00	3.04	2.71	3.02
	*p*-value	0.014	0.001	0.000	0.007	0.036
Year of community establishment	Before 2000	3.23	3.15	3.54	3.46	3.51
	2001–2010	3.35	3.30	3.51	3.57	3.48
	After 2010	3.53	3.47	3.52	3.62	3.53
	*p* value	0.001	0.013	0.418	0.348	0.055

**Table 11:** Kruskal–Wallis test for different age groups.

First-Level Indicators	Second Level Indicators	45–55 Years Old	56–65 Years Old	66–75 Years Old	76 Years Old and Above	*p*-Value
Outdoor environment	Community public facilities	4.00	3.56	3.15	2.89	0.000
	Community sanitation	3.84	3.45	3.01	2.86	0.000
Housing	House decoration	3.29	3.34	2.64	2.24	0.000
	Barrier-free construction and renovation	3.85	3.43	3.31	3.64	0.003
Community services	Home care	3.45	3.42	3.47	3.52	0.000
	Health courses	3.73	3.61	3.10	3.05	0.003
	Medical and health care institutions	3.61	3.35	2.89	3.38	0.001
Social participation	Social activities	3.87	3.74	3.56	2.78	0.000
	Job opportunities	3.71	3.32	2.44	2.33	0.002
Technology to help older adults	Ageing information platform	3.64	3.21	2.76	2.43	0.000
	Intelligent aging products	3.80	3.49	2.79	2.45	0.000

**Table 12:** Results of the Kruskal–Wallis test for different backgrounds.

	Living Arrangements			*p*-Value	Year of Community Construction			*p*-Value
	Living Alone	Living with Spouse	Living with Children		Before 2000	2001–2010	After 2010	
Community public facilities	3.68	3.43	3.32	0.000	3.94	3.60	3.16	0.022
Accessibility of frequently visited places	3.46	3.13	2.94	0.000	3.17	3.19	3.95	0.000
House decoration	3.50	2.95	2.86	0.000	3.14	3.08	2.81	0.000
Convenient facilities inside the room	3.79	3.14	3.08	0.000	3.69	3.33	3.02	0.000
Construction and renovation for better accessibility	4.11	3.49	3.25	0.000	3.68	3.43	3.15	0.000
Medical and health care institutions	3.67	3.29	3.08	0.017	3.37	3.48	3.57	0.000
Social activities	3.60	3.47	3.09	0.000	3.31	3.55	3.84	0.003
Work opportunities	3.78	2.99	2.52	0.000	3.29	3.34	3.47	0.000
Smart product learning	3.19	3.12	2.98	0.000	3.32	3.10	3.47	0.001

## Data Availability

Not applicable.
